# Ongoing neurogenesis in the adult dentate gyrus mediates behavioral responses to ambiguous threat cues

**DOI:** 10.1371/journal.pbio.2001154

**Published:** 2017-04-07

**Authors:** Lucas R. Glover, Timothy J. Schoenfeld, Rose-Marie Karlsson, David M. Bannerman, Heather A. Cameron

**Affiliations:** 1 National Institute of Mental Health, National Institutes of Health, Bethesda, Maryland, United States of America; 2 Department of Experimental Psychology, University of Oxford, Oxford, United Kingdom; Cold Spring Harbor Laboratory, United States of America

## Abstract

Fear learning is highly adaptive if utilized in appropriate situations but can lead to generalized anxiety if applied too widely. A role of predictive cues in inhibiting fear generalization has been suggested by stress and fear learning studies, but the effects of partially predictive cues (ambiguous cues) and the neuronal populations responsible for linking the predictive ability of cues and generalization of fear responses are unknown. Here, we show that inhibition of adult neurogenesis in the mouse dentate gyrus decreases hippocampal network activation and reduces defensive behavior to ambiguous threat cues but has neither of these effects if the same negative experience is reliably predicted. Additionally, we find that this ambiguity related to negative events determines their effect on fear generalization, that is, how the events affect future behavior under novel conditions. Both new neurons and glucocorticoid hormones are required for the enhancement of fear generalization following an unpredictably cued threat. Thus, adult neurogenesis plays a central role in the adaptive changes resulting from experience involving unpredictable or ambiguous threat cues, optimizing behavior in novel and uncertain situations.

## Author summary

The ability to predict whether an experience will end favorably is critical for well-being. Cues associated with specific outcomes can aid in prediction, enabling adaptive behaviors, but cue-outcome relationships are often difficult to learn or inherently ambiguous. Human studies have suggested that the hippocampus, a brain region involved in learning and memory, is also important for predicting outcomes and mediating behavior in situations of uncertainty and conflict. We tested the role of a subtype of hippocampal neurons born in adulthood in responding to ambiguously cued shock. We found that mice without these young neurons show less defensive behavior than normal mice when they hear an ambiguous cue, paired with shock in 50% of trials, but react normally when the cue perfectly predicts the shock. In a novel situation, normal mice behave defensively after ambiguously cued shocks but show very little anxiety-like (defensive) behavior if shocks were predictable. Mice without new neurons fail to make this adaptive change, showing moderate levels of anxiety-like behavior regardless of the predictability of earlier threats. Our findings suggest that an important role for the continued neurogenesis in the hippocampus is to enable adaptive changes to future behavior depending upon predictability of prior threats.

## Introduction

The dentate gyrus in the mammalian hippocampal formation adds new granule neurons throughout life. Despite intense interest in recent years, the precise function of these adult-born neurons is not well understood. Over the past several years, one idea that has gained considerable support is that adult neurogenesis is important for pattern separation, or the ability to discriminate between highly similar cues [1,2]. Mice with disruptions of adult neurogenesis show impairments in fear context discrimination, discriminating spatially close arms in a radial maze, and object investigation tasks when the correct choice is similar to the incorrect choice [3–5]. Such impairments are thought to reflect a deficit in generating or recalling memories due to ineffective encoding of distinct features of cues or contexts that share many perceptual similarities [1,2,6].

New neurons, however, also have effects on emotional behavior. Inhibiting adult neurogenesis prevents the effects of antidepressants on some anxiety- and/or depressive-like (anxiodepressive-like) behaviors and enhances hormonal and behavioral responses to acute stress [7–10]. Notably, the behaviors affected in these studies (novelty-suppressed feeding, grooming latency, and forced swim) are assessed in one-trial tests that contain no explicit role for associative learning or memory, suggesting that these changes in emotional behavior are not caused by impairments in discrimination performance and pattern separation. However, one common feature of both pattern separation tasks and anxiodepressive behavioral tasks is a high degree of ambiguity or conflict. In the emotionality tasks, uncertainty arises from the conflict between possible behavioral choices that could be made in response to novel and ambiguous cues (e.g., approach versus avoid) in potentially threatening situations, while in the aforementioned pattern separation tasks ambiguity arises from the difficulty of discriminating the highly similar cues. Unpredictability, or ambiguity, is in fact a defining feature of stressful or anxiogenic situations [11–13]. We hypothesize that a key role for new neurons may be in resolving or biasing responses to ambiguous information when potential threats generate uncertainty—a possibility that is consistent with a hippocampal role in processing ambiguity or conflict [13–19].

To test this possibility, we investigated the role of adult neurogenesis in partially predictable situations by assessing mice lacking new neurons on an ambiguously cued fear conditioning task. Mice with pharmacogenetic ablation of adult neurogenesis (TK mice) were trained either with a cue that predicted footshock 50% of the time (ambiguous condition) or in a control condition in which the cue perfectly predicted footshock (reliable condition). This paradigm utilizing a partially predictive cue models situations in which a stimulus, e.g., a stranger in a dark alley or a plane overhead in a war zone, sometimes predicts a threat but can also occur without negative consequences. TK mice responded normally to the reliable cue but exhibited reduced defensive behavior, and showed less hippocampal activation in response to ambiguous cues relative to wild-type (WT) mice. They also displayed less anxiodepressive-like behavior 2 d after the shock training with an ambiguous cue but more after the reliable cue. These findings suggest that new neurons enable animals to use information about the predictability of aversive events in order to modulate fear generalization in subsequent novel and ambiguous situations.

## Results

### Inhibition of adult neurogenesis selectively diminishes freezing to an ambiguous cue

Prior reports have indicated that ablation of new neurons has no effect on cued fear conditioning, consistent with a lack of requirement for the hippocampus in this task [7,20–23]. These studies, however, have used cues that are consistently associated with shock and thus fully predictive of the outcome. To test whether responses to ambiguous cue-outcome relationships are dependent on adult neurogenesis, we asked whether mice lacking new neurons show normal freezing behavior in response to cues that are only partially reinforced, i.e., partially predictive of shock.

**Fig 1:**
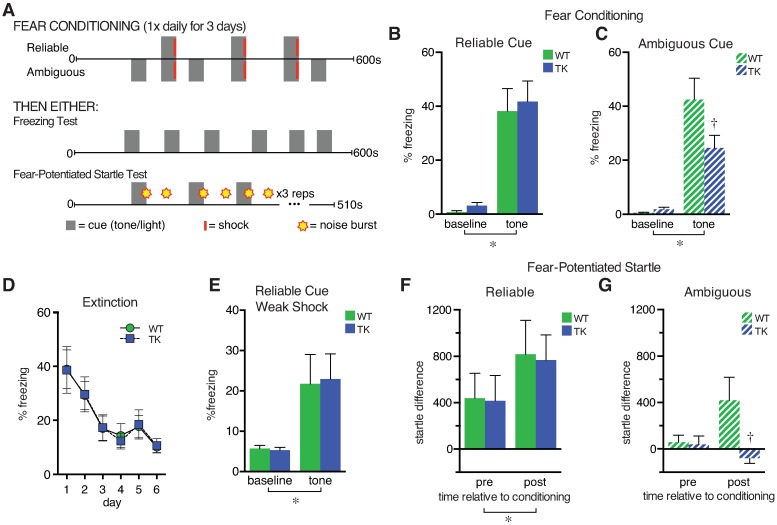
Behavioral response to ambiguous conditioned fear cues is decreased in adult neurogenesis-deficient mice. (A) Examples of conditioned fear training and testing protocols. (B) In a cued fear conditioning task, a perfectly predictive tone cue (Reliable) elicited similar freezing in transgenic (TK) mice (*n* = 8), which lack adult neurogenesis, and wild-type (WT) controls (*n* = 6) (*, main effect of tone versus baseline F_1,12_ = 48.6, *p* < 0.0001; no other significant effects). (C) A tone that coterminated with a shock only 50% of the time (ambiguous) increased freezing in both WT (*n* = 7) and TK mice (*n* = 9; main effect of tone, F_1,14_ = 55.9, *p* < 0.0001; post hoc tests show tone greater than baseline, *p* < 0.005, in both genotypes). However, the tone increased freezing more in WT mice relative to TK mice (tone x genotype interaction, F_1,14_ = 5.0, *p* = 0.04; †, post hoc testing indicates *p* < 0.05 for TK versus WT freezing during the tone). (D) Freezing responses to the reliably predictive tone cue (averaged across six trials for each session) were virtually identical in WT (*n* = 7) and TK (*n* = 8) mice during all extinction days. (E) After reliable cue training with a weak shock (0.3 mA compared to 0.5 mA in earlier experiments), WT (*n* = 11) and TK (*n* = 13) mice showed increased freezing to the tone (main effect of tone F_1,22_ = 13.7, *p* = .001) but equivalent freezing across genotype (main effect of genotype F_1,22_ = 0.007, *p* = .93), suggesting equivalent learning with reliable cues even with a weaker shock training protocol. (F) After fear conditioning, a reliable tone cue increased the startle response similarly in mice of both genotypes (*, main effect of tone F_1,20_ = 4.7, *p* = 0.04, main effect of genotype F_1,20_ = 0.016, *p* = .94; *n* = 11 for both groups). (G) An ambiguous cue increased startle in WT mice (*n* = 11) but not TK mice (*n* = 10) (tone x genotype interaction F_1,19_ = 4.5, *p* = 0.047; †, post hoc testing indicates *p* < 0.05 versus WT at the same time point). Data are represented as mean ± standard error of the mean (SEM). The numerical data used in all figures can be found in S1 Data.

To specifically eliminate adult neurogenesis, we treated 8-wk-old mice expressing herpes thymidine kinase in neuronal precursors with the antiviral drug valganciclovir, which inhibits adult neurogenesis without affecting mature neurons or astrocytes [9]. As expected, adult neurogenesis was virtually eliminated in the dentate gyrus (S1 Fig). Mice lacking adult neurogenesis (TK mice) and WT littermate controls (WT mice) were trained in a 3-day cued fear conditioning paradigm (Fig 1A). In a between-subjects design, half of the mice of each genotype were exposed to a tone cue that always coterminated with a shock (reliable fear conditioning), while the other half were exposed to a tone cue that coterminated with a shock only 50% of the time (ambiguous fear conditioning). Different groups of mice were used for the two cue conditions in order to avoid potential overshadowing or interference between the cues [24,25]. TK mice were indistinguishable from WT controls in response to the reliable cue (Figs 1B and S2), consistent with previous studies [7,20,21,26,27]. However, the TK mice trained with the ambiguous cue showed significantly less cue-induced freezing than their WT counterparts (Figs 1C and S2).

A similar effect was seen in a separate cohort of mice using visual cues as conditioned stimuli, indicating that this effect is independent of the specific sensory modality. Although higher baseline freezing levels were seen after conditioning with light cues as expected [28,29], mice lacking adult neurogenesis froze less than wild types in the ambiguous fear condition but not in the reliable fear condition (S3 Fig). Unconditioned responses to initial presentations of tone, light, and shock were similar in both genotypes, indicating normal sensitivity to the cue and shock in mice lacking adult neurogenesis (S4A–S4D Fig). Freezing in the conditioning (shock) context was low and not different across groups (S4E and S4F Fig), suggesting that both groups attributed predictive salience to the cue. Freezing levels decreased gradually and were well matched for both genotypes across several days of extinction following reliable tone-cued fear conditioning, suggesting that the lack of a deficit in reliable cue fear conditioning was not due to a ceiling effect (Figs 1E and S5). Furthermore, freezing after a reliably cued but weak training protocol using a very mild shock showed no effect of genotype (Fig 1D), suggesting that cue-shock association learning was equivalent across genotypes.

We also measured fear-potentiated startle as an alternative performance measure of fear/anxiety-like behavior, as it may be less prone to contamination by locomotor activity [30]. Separate groups of mice were fear conditioned with a reliable or ambiguous cue, as above, and startle responses were measured both in the presence and absence of the conditioned tones (Fig 1A). The reliable tone cue increased startle responses to the same degree in WT and TK mice (Figs 1F and S6). In contrast, the ambiguous cue increased startle in the WT mice but had no effect in TK mice (Figs 1G and S6), consistent with the findings obtained with freezing measures.

### Loss of adult neurogenesis alters population activity throughout the hippocampus in response to ambiguous threat cues

To better understand how the loss of such a small population of new granule neurons can affect behavior under ambiguous threat conditions, we investigated the impact of new neuron ablation on hippocampal and amygdala network activity during reliably and ambiguously cued shock. Expression of the immediate early gene Fos (c-fos) was analyzed as a measure of neuronal population activity [31–33]. Reliable and ambiguous tone-cued fear training were carried out as above, but mice were perfused shortly after the third training session.

**Fig 2:**
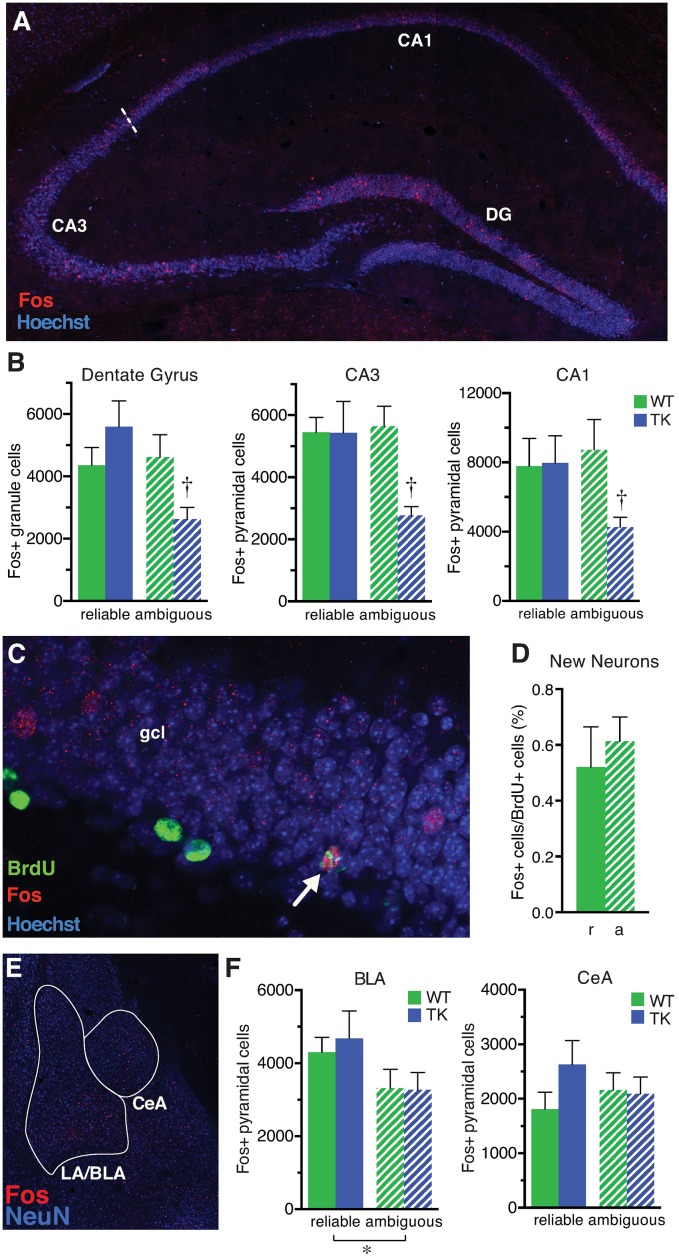
Activation of hippocampal granule and pyramidal neurons by ambiguously conditioned cues is altered in mice lacking adult neurogenesis. (A) Confocal image of the hippocampus shows neurons that were active (Fos+, red) and inactive (blue counterstain) during fear conditioning in the dentate gyrus (DG), CA3, and CA1. (B) Two hours after the third session of tone-shock pairings, TK mice (*n* = 8) had fewer Fos+ cells than wild-type (WT) mice (*n* = 7) in the ambiguous cue condition but not the reliable cue condition (WT, TK *n* = 6, 5) across all hippocampal regions (cue type x genotype interaction F_1,22_ = 6.3, *p* = 0.020; †, post hoc testing indicates *p* < 0.05 versus WT in the same condition/region). (C) Confocal image of Fos immunostaining (red) in BrdU+ (green) cell in the granule cell layer (gcl) shows a 4-wk-old granule neuron active during fear conditioning. (D) Adult-born granule neurons, labeled with BrdU, in WT mice were similarly activated by reliable (r) and ambiguous (a) cue training (t_10_ = 1.1, *p* = 0.32); TK mice had no new neurons. (E) Confocal image of the amygdala shows Fos staining in the lateral/basolateral (LA/BLA) and central (CeA) nuclei of the amygdala. (F) The number of Fos+ LA/BLA cells was lower in the ambiguous cue condition relative to the reliable cue condition but there was no effect of genotype (main effect of predictor type F_1,22_ = 5.0, *p* = 0.0363; main effect of genotype F_1,22_ = 0.09, *p* = .7628; WT, TK *n* = 6, 5). No significant differences were observed across cue type or genotype in the CeA (all effects F_1,22_ < 1.67, *p* > 0.2). Data are represented as mean ± standard error of the mean (SEM). The numerical data used in all figures can be found in S1 Data.

In WT mice, there was no effect of training condition on hippocampal neuron activation; equivalent numbers of Fos-expressing neurons were seen after reliable and ambiguous shock training in the overall granule cell population, new granule neurons, CA3 pyramidal neurons, and CA1 pyramidal neurons (Fig 2A–2D). TK mice, however, had fewer activated mature granule cells and fewer activated CA3 and CA1 pyramidal cells in the ambiguous fear condition relative to the reliable fear condition, resulting in a statistically significant genotype x cue type (reliable/ambiguous) interaction across all three hippocampal subfields (Fig 2B). Similar effects were observed in both the dorsal and ventral subregions of the hippocampus (S7 Fig). There were no significant genotype differences in total freezing behavior across the entire session on the last training day, immediately prior to perfusion (S8 Fig), suggesting that the observed differences in hippocampal neuron activation were unlikely simply to reflect a direct readout of the preceding behavioral performance (locomotor activity levels) of the animals. Within the basolateral nucleus of the amygdala (Fig 2E), the number of Fos-expressing cells did not differ across genotypes, but fewer Fos-expressing cells were seen after ambiguous conditioning compared to reliable conditioning in both genotypes (Fig 2F). No significant differences were found in the central nucleus (Fig 2F). Taken together, these findings indicate that the new neurons influence the wider hippocampal network activation under conditions of ambiguous threat and suggest that the hippocampus, but not the amygdala, contributes to the behavioral changes observed in TK mice in response to ambiguously cued shock.

### Unpredictable shock affects future defensive behavior in a novel environment

**Fig 3:**
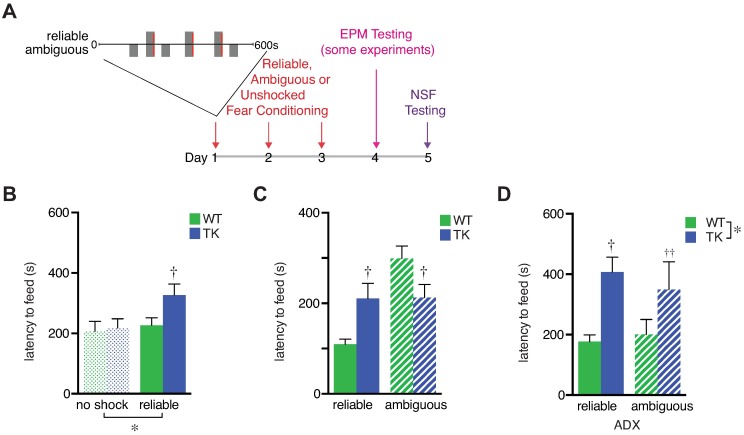
Fear conditioning effects on future behavior depend on cue reliability, adult neurogenesis, and adrenal hormones. (A) Fear conditioning and anxiodepressive behavior testing timeline. (B) Latency to eat in the novelty-suppressed feeding (NSF) test was increased by reliably cued fear conditioning in TK mice (*n* = 20), but not wild-type (WT) mice (*n* = 19), relative to unshocked mice of both genotypes (WT, TK *n* = 19, 19; training effect F_1,73_ = 4.1, *p* = 0.048; genotype effect F_1,73_ = 3.0, *p* = 0.086; interaction F_1,73_ = 2.0, *p* = 0.165; †, post hoc testing indicates *p* < 0.05 versus unshocked condition. (C) NSF latency was increased by ambiguous cue training, relative to reliable cue training, in WT mice but not TK mice. WT mice had longer latencies than TK mice after ambiguous cue training but had shorter latencies than TK mice after reliable cue training (predictor type x genotype interaction F_1,77_ = 12.3, *p* = 0.0008; †, post hoc testing indicates *p* < 0.05 versus WT in the same condition; WT *n* = 20, 21; TK *n* = 20, 20 for reliable, ambiguous). (D) When mice were adrenalectomized, latency to eat was longer in TK mice than WT mice regardless of cue type (*, genotype main effect F_1,25_ = 11.5, *p* = 0.002; †, post hoc testing indicates *p* < 0.05; ††, *p* < 0.1 versus WT in the same condition; WT *n* = 7,7; TK *n* = 9, 6 for reliable, ambiguous). Data are represented as mean ± standard error of the mean (SEM). The numerical data used in all figures can be found in S1 Data.

We next asked whether exposure to reliable or ambiguous tone-shock relationships influences subsequent behavior in novel situations and whether new neurons play a role in such adaptive responses. To do this, we assessed novelty-suppressed feeding (NSF) behavior, a hippocampus-dependent test of anxiodepressive-like behavior [34], in a novel setting 2 d after fear conditioning (Fig 3A).

In mice with normal levels of adult neurogenesis, exposure to reliable shock had no effect on subsequent NSF latency relative to a control group that experienced tones with no shocks (Fig 3B). In contrast, reliably cued fear conditioning increased latency to feed in mice lacking adult neurogenesis (Fig 3B). Thus, although the WT and TK mice had similar conditioned freezing responses (and hippocampal activation) to the reliable conditioned cue (Figs 1 and 2), they later showed differences in behavior when tested in a novel situation, with TK mice failing to suppress anxiodepressive-like responses.

NSF behavior in a separate cohort of mice, which underwent fear conditioning using either the reliable or ambiguous cues, showed a genotype x predictor type interaction. WT mice took longer to eat after being conditioned with the ambiguous cue relative to mice trained with the reliable cue (Figs 3C and S9). TK mice, on the other hand, were unaffected by cue predictability, as demonstrated by their similar latencies following reliable or ambiguous cue training. The TK mice exhibited longer latencies than WT mice after reliable fear conditioning (as in the previous experiment shown in Fig 3B) yet shorter latencies than WT mice after ambiguous fear conditioning (Fig 3C). These findings demonstrate that normal mice utilize information about the predictability of prior threat to adapt their level of cautious, or anxiodepressive-like, behavior in future situations. Mice without adult neurogenesis increase anxiodepressive-like behavior as a result of being shocked, but they do so without regard to the predictability of the shock. Taken together, these data suggest that new neurons have a bidirectional adaptive effect, suppressing neophagia in novel contexts following predictable shock while enhancing cautious behavior in new situations following ambiguous threat.

Behavior was also tested in the elevated plus maze (EPM), but no effects of cue predictability or genotype were observed (S10 Fig). The key difference between the NSF and EPM tests is unclear, but previous studies have also found that even though both tests are sensitive to hippocampal lesions [34,35], the EPM is less sensitive to changes in adult neurogenesis [7,9,26] (but see [36]).

### Increased anxiodepressive-like behavior following unpredictable cued shock requires glucocorticoids as well as new neurons

Glucocorticoids enhance fear/anxiety in a strong shock model of posttraumatic stress disorder and play an important role in adaptive matching of stress resilience to the developmental environment [37,38]. We therefore asked whether these stress hormones also mediate the adaptive changes observed here following unpredictable shock in ambiguous cue fear conditioning. To do this, we assessed NSF in mice that were adrenalectomized prior to fear conditioning, preventing stress-induced glucocorticoid release. In these mice, we found increased latency to eat in TK mice relative to WTs, regardless of predictor type (Fig 3D). Compared with the previous, adrenal-intact, experiment (Fig 3C), the primary difference appears to occur in the WT mice trained with the ambiguous cue, which failed to show the strong increase in NSF latency after adrenalectomy. This finding indicates that glucocorticoids produced during fear conditioning (S11 Fig) can increase future anxiogenic (neophagic) behavior in novel situations, but only after ambiguously cued shocks and only in animals with new neurons. Loss of adrenal stress hormones had no effect on mice trained in the reliable cue condition, consistent with the idea that unpredictability is a critical feature of stress [11].

## Discussion

Here, we show that neurons born in the adult hippocampus enable bidirectional adaptive changes in defensive behavior under conditions of ambiguously cued threat. Control mice, with normal levels of adult neurogenesis, freeze similarly to a cue that reliably predicts shock and to a partially predictive (ambiguous) cue. In a novel environment without shock-associated cues, control mice that experience predictable shocks show no increase in feeding latency, an anxiodepressive-like behavior, relative to unshocked mice. However, normal mice that received the same number of shocks, but in a less predictable manner with respect to the conditioned stimulus, show a strong glucocorticoid-dependent increase in their feeding latency. When mice lacking adult neurogenesis are trained with the ambiguous cue, they show less defensive behavior than controls in three different tests: decreased freezing to the cue following fear conditioning, decreased startle in the presence of the cue following fear conditioning, and decreased latency to eat during the NSF test in a novel environment. However, following reliably cued shock, mice without adult neurogenesis exhibit greater neophagia than normal mice in a novel context. Taken together, these findings suggest that defensive behavior in mice following an adverse experience reflects a combination of processes: (i) an increase in defensive behavior resulting from ambiguity in cues predicting threat (similar to a stress response), which is mediated by adult neurogenesis and glucocorticoids, and (ii) inhibition, or contextualizing, of fear/anxiety following a reliably cued threat, which also requires adult neurogenesis but is glucocorticoid-independent.

Strikingly, the changes in defensive behavior following reliably and ambiguously cued shock were paralleled by changes in neuronal activation in the dentate gyrus, CA3, and CA1. This finding, i.e., decreased activation throughout the hippocampus in TK mice in response to an unreliable predictor of threat (ambiguous cue), suggests that adult-born granule neurons normally recruit additional granule neurons and pyramidal neurons under partially predictable threat conditions. Adult-born granule cells likely increase hippocampal activity via direct synaptic connections with CA3 pyramidal cells [39] and disynaptic connections with CA1 pyramidal cells via CA3 Schaffer collaterals. Hilar mossy cells provide a possible link between new granule cells and mature granule cell activity [39,40]. Previous studies have described the excitability of adult-born neurons [41–43], but the role of adult neurogenesis on hippocampal networks has remained unclear, with competing hypotheses suggesting that new neurons preferentially excite or inhibit the dentate gyrus and CA3 [40]. The current findings support the idea that new granule cells excite the hippocampus, in contrast to the preferential inhibition predicted by a role in sparse encoding/pattern separation or observed in slice physiology experiments [40]. However, the bidirectional effects we observe in behavior suggest that adult-born granule cells could potentially also diminish activity in the hippocampal network under certain circumstances, e.g., in novel situations following reliably cued threat, although this has not been tested.

The results of this study and others suggest an association between hippocampal activation and increased fear/anxiety. In one study, more ventral CA1/subiculum neurons are activated following a conditioned fear cue compared to an extinguished cue [44]. Similarly, more granule neurons are activated in rats during swimming, either in a water maze learning task or in a control condition lacking a platform, than under cage control conditions [42]. Consistent with these earlier findings, the treatment group showing the lowest level of freezing to the tone on the test day in the current study, the TK mice trained with the ambiguous cue, had the fewest activated granule neurons and pyramidal neurons on the prior day. The only previous study to look at fear-related neuronal activity in mice lacking adult neurogenesis found that irradiated mice had more activated mature granule cells than intact mice in an active place avoidance task on a rotating platform [45], which appears inconsistent with the decreased granule cell activation in TK mice in the current study. However, in the current study all mice received the same number of shocks, whereas in the place avoidance study the mice without new neurons received more shocks than control mice, so their increased granule cell activation is also consistent with a positive relationship between hippocampal activation and fear. No previous studies have looked for changes in experience-induced neuronal activation outside the dentate gyrus associated with loss of adult neurogenesis. The decreased recruitment of neurons throughout the hippocampus observed here may explain how loss of a relatively small number of adult-born granule cells [21,46] can have significant effects on behavior.

The effects of new neuron ablation on hippocampal network activation in the current study did not extend to the amygdala, which therefore seems unlikely to drive the behavioral changes observed in TK mice relative to wild types. Under reliable threat conditions, the hippocampus is strongly activated, with or without new neurons, but this activation may be unnecessary or redundant, as the hippocampus is usually not required for normal behavior when a straightforward cue-shock relationship exists [22,47]. Under conditions of ambiguous threat, however, the decreased freezing seen in TK mice may reflect the decreased lateral/basolateral amygdala activation in this condition seen in both genotypes. In normal WT mice, decreased amygdala activation may be offset by activation of young granule neurons, leading to enhanced activation throughout the hippocampal network and a resulting increase in behavioral inhibition and thus greater freezing [13].

Decreased freezing behavior in TK mice in response to the ambiguous cue could in principle reflect impaired fear learning, but several observations argue against an associative learning deficit. First, the TK mice showed normal fear conditioning with a reliable cue, even in the more difficult version of the task using a very weak shock, and showed equivalent freezing levels to controls throughout extinction of reliable cue fear conditioning. Both of these findings argue against the masking of a learning impairment by a ceiling effect. Second, although freezing levels were different in the ambiguous condition, TK mice significantly increased freezing in the presence of the cue relative to baseline and pre-cue time points, indicating that they learned the cue-shock association. Previous work found that levels of conditioned freezing are determined by a relatively fixed associative learning component and a highly variable nonassociative component [48], suggesting that differential freezing in the current study reflects a change other than the strength of associative learning. Third, contextual fear was low in TK mice, suggesting that they, like WT mice, attributed greater weight or salience to the cue as a predictor of the shock [24,25,49]. This was true under ambiguous as well as reliable conditions. Finally, the behavioral changes in the NSF test are unlikely to reflect impaired associative learning, as this test involved no explicit role for conditioned stimuli, i.e., no specific cue or context to guide learned behavior.

Because the fear conditioning tasks in our study utilized one single cue, which was exactly the same in shock and no-shock trials, the behavioral effects in TK mice cannot be explained by an impairment in the discrimination of highly similar cues [2], as in “pattern separation” tasks described previously [3–5]. However, it is possible that the observed changes in freezing and startle behavior to the ambiguous cues in the TK mice reflect an impairment in “pattern separation” or disambiguation at the level of retrieval of overlapping tone-shock and tone-no shock associative memories or, similarly, the meaning behind the tone (tone-safety and tone-danger). A related possibility is that TK mice are impaired in choosing between the competing behavioral responses associated with those memories (tone-freeze versus tone-don’t freeze) [15,50]. Performance impairments resulting from response competition, in the absence of memory impairment, have also been described in partial reinforcement spatial tasks performed under stressful conditions [51–53].

The adult neurogenesis-dependent effects on neophagia most likely reflect a form of fear generalization. The parallel results across experiments suggest that the enhancement of freezing and startle in normal mice may also reflect the same process. Such generalization is critical to survival, as it enables an organism to enhance alertness and quickly respond to threats not specifically encountered before [54]. It is frequently viewed as being driven by perceptual similarity to previously encountered threat cues (i.e., stimulus generalization). Recent evidence from human studies, however, supports an alternative to this perceptual model in which fear generalization is instead driven by an active process triggered by ambiguity in threat outcome, which is then integrated with passive cue similarity information [12]. This view of generalization as an active ambiguity-driven process fits with our findings that both stress hormones and signals from new neurons enhance fear generalization following ambiguous, potentially threatening events. The hippocampus has previously been implicated in fear generalization, a role variously described as supporting pattern separation of perceptually similar cues to minimize stimulus-based fear generalization [55] or as biasing behavior during uncertain expectation by strengthening the representation of aversive potential outcomes [13,56]. The current findings support a hippocampal role in ambiguity-based fear generalization and suggest an important role for ongoing neurogenesis, in particular, possibly in predicting or weighting possible outcomes associated with different behavioral options [57,58] and/or in emotional biasing of decisions [13,16,56,59].

Fear generalization in response to severe threat, a possible model for posttraumatic stress disorder, occurs even when shock is reliably cued and is known to rely on plasticity in a population of neurons in the lateral amygdala [48,54]. A recent study found that nonassociative, or generalization, effects of strong shock are enhanced in mice lacking new neurons [36], consistent with enhancement of stress response in these animals [9]. The persistent behavioral effects of strong shock, i.e., increased freezing in shock-associated and/or novel contexts, are not observed in mice exposed to mild shock [36]. The current study demonstrates that fear generalization can also occur in response to mild shock if it is ambiguously cued. The relationship between fear generalization induced by strong threat and by ambiguously cued threat is unclear; differences in the behavioral changes observed by Seo et al. and in the current study could indicate that these two forms of fear generalization are entirely distinct, or they may simply reflect differences in fear intensity.

Conceptually, fear generalization might describe a transition from fear, the emotional response to a specific stimulus that indicates that danger is imminent, to anxiety, a longer-lasting emotional state generated by less specific, less predictable, or potential threats [13,60,61]. Fear generalization is highly adaptive, yet hyperactivity in fear generalization circuits may drive excessive or inappropriate fear responses, which can limit more rewarding actions and lead to generalized anxiety disorder [60,62,63]. Too little generalization can be equally detrimental, posing a threat to survival or welfare by failing to promote active defensive behaviors and avoidance of danger. Thus, there is an optimal level of fear generalization, resulting in a balance between competing approach and avoidance behaviors [63,64], but exactly where this balance lies is specific to a given situation. The current results suggest that adult hippocampal neurogenesis plays a role in continually and flexibly optimizing this balance based on prior experience.

The adaptive nature of fear generalization has been highlighted in the “predictive adaptive response hypothesis” [65,66], which suggests that the level of adversity in the developmental environment biases protective stress or anxiety responses in adulthood to better adapt an organism to the environment into which it is born. The current findings suggest that adult neurogenesis, through a role in predicting or biasing outcomes of ambiguous events, allows for this type of matching between the environment and the level of fear generalization to continue into adulthood [67]—in effect extending a behavioral form of developmental plasticity. By enhancing or inhibiting fear generalization according to the predictive ability of environmental cues, changes in the production of new neurons can potentially affect behavior in any situation featuring ambiguity generated by a potential threat or, more broadly, any difficult choice [11,12,57]. The current findings therefore provide a potential link between the roles of new neurons in the stress response, anxiodepressive-like behavior, and pattern separation.

## Methods

### Animals

Transgenic male mice (TK mice) expressing the herpes simplex virus thymidine kinase under the human glial fibrillary acidic protein promoter and maintained on a CD-1 background [9] and WT littermate controls were generated from heterozygous x WT matings, weaned at 3 wk of age, genotyped via PCR, and housed 3 to 4 per cage with mixed genotype siblings. Beginning at 8 wk of age, mice were treated with valganciclovir p.o. (0.3%, 35 mg/kg/d), 4 d/wk, for 8–9 wk before behavior testing. Mice were housed under a 12-h light:dark cycle, and all testing took place during the dark phase. All procedures were approved by the NIMH Animal Care and Use Committee and comply with NIH guidelines (PHS Animal Welfare Assurance A4149-01).

### Handling and general procedures

Mice were identified by randomly assigned ear tag numbers so investigators were blind to genotype. Mice were handled 3–5 min/d for 3 d prior to behavioral testing and were brought to a dark holding area 30 min prior to testing on each day.

### Cued fear conditioning

Fear conditioning was conducted in a clear-walled, 30 x 30 x 24 cm chamber (Coulbourn Instruments), which was cleaned with 70% EtOH after each session. The unconditioned stimulus (US) was a 0.5-mA (except in the weak shock experiment, where it was 0.3 mA), 1-s scrambled shock delivered through the grid floor. Cues (Coulbourn bright light or 2 kHz, 85 dB(A) tone) lasting 20 s served as conditioned stimuli. Mice in the “reliable” groups received three cue-shock pairings per session, with the cue always coterminating with a shock. Mice in “ambiguous” groups received the same three cue-shock pairings and three additional cue-only trials in each session, so the cue coterminated with a shock in only 50% of trials. Sessions lasted 600 s with a 120-s habituation period prior to cue presentation for tone fear conditioning (660 s with 180-s habituation for light fear conditioning). To look at IEG expression, a separate cohort of mice received tone-cued fear conditioning but were perfused 2 h after the third training day for histological analysis (see below).

Mice were trained in 3 sessions separated by 24 h. The timing of cues, and order of cue-shock and cue-only trials for the ambiguous group, was pseudorandom and differed across consecutive days. Freezing to the conditioned tone (or light) was tested in a novel arena 24 h after the last training day. Freezing during six 20-s cue presentations was compared with freezing during the 100-s baseline prior to the first cue. Activity response to the first shock was analyzed using TopScan (CleverSys, Inc). For the reliable auditory cue fear conditioning groups, six daily extinction sessions, each with six 20-s tones and no shocks, beginning 1 d after cued fear recall testing, were given back in the original training context. Freezing during the first 2 min (habituation) of the first extinction day served as a measure of contextual freezing. Context freezing was analyzed following cued fear conditioning in the light conditioning experiment. Freezing was analyzed using FreezeView software (Coulbourn Instruments).

### Fear-potentiated startle

Fear conditioning and startle testing were conducted in acoustic startle boxes (Med Associates Inc.) in naïve mice. The 9-d experiment included a habituation day, 2 noise burst intensity test days, a rest day, a preconditioning test, 3 d of “reliable” or “ambiguous” fear conditioning, and a postconditioning test, following a published protocol [68]. Reliable and ambiguous groups were run during consecutive weeks.

During preconditioning, startle to noise burst alone (NBA) and noise bursts preceded by an unconditioned tone (TNB) served as a baseline. Tones were 20 s, 2 kHz, and 70 dB(A). After a 2-min acclimation period, noise bursts (75, 80, and 85 dB(A)) were presented 3 times alone (9 trials) in a pseudorandom order, followed by NBA trials intermixed with TNB trials. An internal chamber fan (63 dB) was used to mask external sounds throughout the startle experiment. All ITIs were 30 s.

For fear conditioning, the same protocol as the tone-cued fear conditioning experiments was used, except the shock was 250 ms in duration (0.5 mA). Startle was tested using the same protocol as in preconditioning (above), except with variable ITIs ranging between 80–120 s (reliable conditioning group) or 50–70 s (ambiguous conditioning group). Different variable ranges were used in order to keep time in the chambers the same between each group. Peak-to-peak startle amplitudes were collected 600 ms following the noise burst, and the average of the difference scores from the 75 and 80 dB(A) trials were used.

### NSF

Mice were tested in a white arena (50 x 50 x 40 cm) with bedding just covering the floor. One pellet of familiar food was placed on top of a 1-cm-high white dish in the center of the arena where light was set to 290 lux. Latency to begin eating the food was scored (maximum trial time of 10 min).

### EPM

This maze had four arms, each 50 x 10 cm, two of which had 40 cm high walls (closed arms), and a 10-cm^2^ center zone. The maze was elevated 50 cm above the ground, surrounded by black curtains, illuminated to 700 lux, and cleaned with 70% EtOH between trials. Mice were allowed to explore freely for 5 min. Time spent in each arm was determined using TopScan (CleverSys, Inc.).

### Corticosterone manipulation and measurement

To clamp corticosterone levels, adrenal glands were removed from mice under isoflurane anesthesia 5 d prior to behavior. Adrenalectomized mice were given saline (0.9% NaCl) with low-dose corticosterone replacement (25 μg/ml with 0.15% v/v ethanol) in drinking water to maintain baseline levels of corticosterone [69]. After behavior testing, corticosterone replacement was discontinued for 4 d, blood was sampled under isoflurane, and serum corticosterone was measured via radioimmunoassay (MP Biomedicals). Two mice were excluded due to incomplete adrenalectomy (levels >40 ng/ml). To measure corticosterone responses to fear conditioning in adrenal-intact mice, submandibular blood was sampled from unanesthetized mice 30 min after fear conditioning (ambiguous and reliable), and serum corticosterone levels were measured as above.

### Histology

To confirm ablation of neurogenesis, brains from all mice were fixed in 4% paraformaldehyde, sectioned, and stained with anti-doublecortin (Santa Cruz Biotech, sc-8066) [9].

To identify adult-born neurons in the IEG experiment, mice were given BrdU (1 mg/mL, Roche; with 1% sucrose) in drinking water for 6 d and underwent tone-cued fear conditioning 4 wk after BrdU treatment began. Mice were perfused with 4% paraformaldehyde 2 h after the start of the third training session. Brains were sectioned at 40 μm through the entire rostral-caudal extent of the hippocampus.

For IEG analysis, 1:8 series of sections were triple-stained using 2 h denaturation in 2N HCl and 3-day incubation in rat anti-BrdU (1:200; Accurate Chemical OBT0030), goat anti-c-fos (1:250; Santa Cruz Biotechnology sc-52-G), and mouse anti-NeuN (1:250; Chemicon MAB377) [70]. Staining was visualized with anti-rat Alexa 488, anti-goat Alexa 555, and anti-mouse Alexa 633 (all 1:200, Life Technologies A21208, A21432, and A31571, respectively). Sections were mounted, coverslipped with PermaFluor (Thermo Scientific), and coded prior to analysis.

Total counts of Fos+ cells in the dorsal and ventral dentate gyrus granule cell layer dorsal and ventral CA3 and CA1 pyramidal cell layer, and basolateral and central amygdala in stained series were multiplied by the series interval to obtain stereological counts. All BrdU+ cells in the granule cell layer were counted and examined for colabeling with Fos, using optical stacks of 1 μm confocal sections and examination of orthogonal planes (Olympus FV300, 60X) to confirm double-labeling. One mouse was excluded from BrdU analysis because it did not have enough BrdU+ cells to meet the criterion of 100 cells analyzed. Another data point was excluded because it was >3 SD higher than the mean; removal of this point did not affect the outcome of the test.

### Statistics

Experiments were run on mixed genotype litters born in the same week (cohorts), pseudorandomly assigned by cage to treatment conditions. Data were analyzed with GraphPad Prism and SPSS software. All *t* tests were independent samples (two-tailed). All other comparisons were two-way or three-way mixed or between-subjects ANOVA, as appropriate. All post hoc comparisons were made with corrections for multiple comparisons.

## Supporting information

S1 Fig Quantification of neurogenesis in GFAP-TK mouse model. Valganciclovir (VGCV) treatment eliminates BrdU-labeled new neurons from the dentate gyrus of TK mice but not WT mice (*, t_12_ = 6.2, *p*<0.0001). Data are represented as mean ± SEM.
(PDF)

S2 Fig Trial-by-trial data for fear conditioning with a tone cue. Freezing data are shown for individual trials across the 3 training days and test day for mice trained under reliable and ambiguous cue conditions. Freezing at baseline each day, prior to the first tone, during the tone (gray bars) and 20-sec pre-tone periods are shown. Red lines symbolize shocks that occur at the end of every training trial in the reliable condition and half of the trials in the ambiguous condition; no shocks occurred on the test day. Data are represented as mean ± SEM.
(PDF)

S3 Fig Ambiguous fear conditioning with a light cue. (A) Fear conditioning with a reliable light cue had a similar effect on both genotypes (*, light effect F_1,15_ = 18.4, *p* = 0.0007). TK mice froze less than WT mice following ambiguous cued fear conditioning with a light cue (*, light effect F_1,15_ = 10.4, *p* = 0.006; genotype effect F_1,15_ = 9.1, *p* = 0.009; †, post hoc testing indicates *p*< 0.05 versus WT at the same time point and condition). In a 3-way ANOVA with cue modality (light/tone), genotype, and predictor (reliable/ambiguous) as factors, the genotype x predictor interaction was significant (F_1,56_ = 4.6, *p* = 0.036), with TK mice freezing less than WT mice only after ambiguous conditioning. (B) Freezing data are shown for individual trials across the 3 training days and test day for mice trained under reliable and ambiguous cue conditions. Freezing at baseline each day, prior to the first cue, during the light cues (yellow bars), and during the 20-sec pre-cue periods are shown. Red lines symbolize shocks as in S2 Fig. Data are represented as mean ± SEM.
(PDF)

S4 Fig Unconditioned responses to fear conditioning stimuli. (A) Unconditioned freezing during the first presentation of the tone in tone fear conditioning was similar in WT and TK mice (period, i.e., pre-tone vs. tone, effect F_1,27_ = 4.9, *p* = 0.036; no other significant effects). (B) Unconditioned freezing was also similar in WT and TK mice during the first light presentation in fear conditioning to light (period effect F_1,33_ = 249.4, *p*<0.0001; no other significant effects). (C) Motor/bursting activity during the first shock did not differ across genotype during tone fear conditioning (time effect: F_2,54_ = 83.9, *p*<0.0001; no other significant effects), suggesting similar shock sensitivity. (D) Motor/bursting activity during the first shock also did not differ across genotype during light fear conditioning (time effect: F_2,66_ = 198.6, *p*<0.0001; no other significant effects). (E) When tone fear conditioned mice were placed back into the original training context, without cues or shocks, mice in both genotypes showed similar low freezing scores (no significant main effects or interaction; # indicates main effect of predictor type F_1,27_ = 3.7, *p* = 0.0654). (F) When light fear conditioned mice were placed back into the original training context, but without cues or shocks, mice in both genotypes showed similar low freezing scores (no significant main effects or interaction). Data are represented as mean ± SEM.
(PDF)

S5 Fig Trial-by-trial data for extinction of cued fear. Freezing data are shown for individual trials across the 6 days of extinction following reliable cue training/testing. Freezing during baseline (BL) prior to the first tone, during the tones (gray bars), and 20-sec pre-tone periods are shown for each trial. Data are represented as mean ± SEM.
(PDF)

S6 Fig Raw data for fear-potentiated startle. Startle amplitude in arbitrary units (a.u.) is shown for noise burst alone trials (NBA) and for trials in which noise bursts are preceded by the tone cue (TNB). The difference between NBA and TNB reflects potentiation of fear by the cue before fear conditioning (no outline) and after fear conditioning (red outline) with the reliable or ambiguous protocol. In the cohort trained with the Reliable cue, the tone cue increased startle relative to the NBA both pre- and post-conditioning, with no effect of genotype (*, main effect of tone pre: F_1,19_ = 6.4, *p* = 0.02, post: F_1,19_ = 16.3, *p* = 0.0007; main effect of genotype pre: F_1,19_ = 0.08, *p* = 0.78; main effect of genotype F_1,19_ = 0.004, *p* = 0.95). In the cohort trained with the Ambiguous cue, there were no significant main effects or interactions prior to conditioning. After conditioning with an ambiguous tone cue, a tone x genotype interaction (F_1,19_ = 5.4, *p* = 0.0308; †, post hoc testing indicates *p*<0.05 versus WT NBA and TK TNB) shows that the tone increased startle in the WT mice but not TK mice. Data are represented as mean ± SEM.
(PDF)

S7 Fig Neuronal activation by fear conditioning in dorsal and ventral hippocampus. (A) and (B) IEG expression patterns were similar in the dorsal and ventral portions of the dentate gyrus, with fewer Fos+ granule cells in TK mice than WT mice in the ambiguous cue condition but not the reliable cue condition (cue type x genotype interaction: F_1,21_ = 6.9, *p* = 0.02 in the dorsal dentate gyrus and F_1,21_ = 4.7, *p* = 0.04 in the ventral dentate gyrus; †, post hoc testing indicates *p*<0.05 relative to WT in the same condition). A three-way ANOVA with cue type, genotype, and region as factors showed a significant cue type x genotype interaction (F_1,22_ = 6.82, *p* = 0.02) but no cue type x genotype x region interaction (F_1,22_ = 2.08, *p* = 0.16). (C) and (D) IEG expression patterns were also similar in dorsal and ventral CA3, with fewer Fos+ pyramidal cells in TK mice than WT mice after ambiguous, but not reliable, fear conditioning (cue type x genotype interaction: F_1,21_ = 4.6, *p* = 0.04 in the dorsal CA3 and F_1,21_ = 3.9, *p* = 0.06 in the ventral CA3; †, post hoc testing indicates *p*<0.05 relative to WT in the same condition). A three-way ANOVA with cue type, genotype, and region as factors showed a significant cue type x genotype interaction (F_1,22_ = 5.70, *p* = 0.026) but no cue type x genotype x region interaction (F_1,22_ = 0.06, *p* = 0.80). (E) and (F) In the dorsal CA1, TK mice had fewer Fos+ pyramidal cells than WT mice after ambiguous, but not reliable, fear conditioning (cue type x genotype interaction: F_1,22_ = 3.5, *p* = 0.07; †, post hoc testing indicates *p*<0.05 relative to WT in the same condition). In ventral CA1, Fos expression did not show significant main effects (cue type: F_1,22_ = 1.3, *p* = 0.27, genotype: F_1,22_ = 1.1, *p* = 0.31) or a cue type x genotype interaction (F_1,22_ = 1.6, *p* = 0.22; †, post hoc testing indicates *p*<0.05 relative to WT in the same condition), but the pattern appeared similar to that in the dorsal CA1. A three-way ANOVA with cue type, genotype, and region as factors showed no cue type x genotype interaction (F_1,22_ = 2.74, *p* = 0.11) but a trend toward a cue type x genotype x region interaction (F_1,22_ = 4.13, *p* = 0.054).
(PDF)

S8 Fig Freezing behavior during fear conditioning session prior to IEG measurement. Freezing behavior during the entire 10 min session on the last day of fear conditioning training 2 hr prior to sacrifice was not significantly different across genotype or predictor type, suggesting that differences in behavior during this session did not drive changes in Fos expression. Data are represented as mean ± SEM.
(PDF)

S9 Fig Novelty-suppressed feeding data shown as a survival curve. Latency to feed in a novel environment, plotted as mice that have fed at each time point, shows significant differences across genotype/treatment groups (Mantel-Cox test, X^2^ = 6.255, *p* = 0.0124). Most mice ate prior to the cutoff of 600s, suggesting that assumptions of normal distribution are not violated, and two-way ANOVA can be used for further analysis.
(PDF)

S10 Fig Behavior on the elevated plus maze. (A) In experimentally naive mice, there was no effect of loss of adult neurogenesis on the percentage of time in open arms of the elevated plus maze. (B) The same mice showed no difference in average velocity during maze exploration. (C) After fear conditioning, a separate cohort of mice showed no effect of predictor type (reliable/ambiguous) or genotype on the percent of time in open arms. (D) The same fear conditioned mice showed no differences in average velocity during exploration. Data are represented as mean ± SEM.
(PDF)

S11 Fig Corticosterone release in response to fear conditioning. Mice trained on reliable and ambiguous cue fear training both show increased serum corticosterone 30 min after fear conditioning, relative to baseline animals, which had only two days of ambiguous fear conditioning and were tested directly after removal from their home cage (conditioning type: F_2,42_ = 16.7, *p*<0.0001; †, post hoc testing indicates *p*<0.05 relative to treated groups; #, post hoc testing indicates *p* = 0.0732).
(PDF)

S1 Data Complete dataset. Excel spreadsheet containing the individual data points underlying analyses and graphs shown in all figures.
(XLSX)

## Abbreviations

EPM: elevated plus maze
NSF: novelty-suppressed feeding
TK mice: mice with pharmacogenetic ablation of adult neurogenesis
WT: wild-type
