# Correction: Ongoing neurogenesis in the adult dentate gyrus mediates behavioral responses to ambiguous threat cues

**DOI:** 10.1371/journal.pbio.1002604

**Published:** 2017-05-09

**Authors:** The PLOS Biology Staff

## Notice of Republication

This article was republished on April 14, 2017 to correct some textual errors that were introduced during the typesetting and proof processes. The publisher apologizes for the errors. Please download this article again to view the correct version. The originally published, uncorrected article and the republished, corrected articles are provided here for reference.

## Supporting information

S1 File Originally published, uncorrected article. (PDF)

S2 File Republished, corrected article. (PDF)
