# Supplementary figures and images for: Ongoing neurogenesis in the adult dentate gyrus mediates behavioral responses to ambiguous threat cues
Source: PLoS Biol. 2017 Apr 7;15(4):e2001154. doi: 10.1371/journal.pbio.2001154 (PMC5384657; doi:10.1371/journal.pbio.2001154)

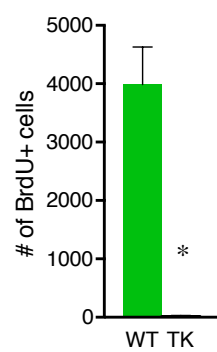

Supplement: S1 Fig — Valganciclovir (VGCV) treatment eliminates BrdU-labeled new neurons from the dentate gyrus of TK mice but not WT mice (*, t12 = 6.2, p<0.0001). Data are represented as mean ± SEM. (PDF) [file pbio.2001154.s001.pdf]

## A Reliable

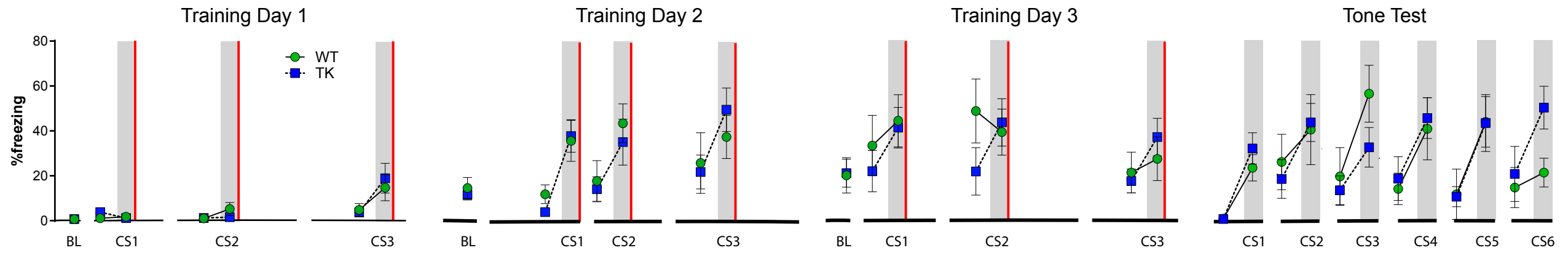

## B Ambiguous

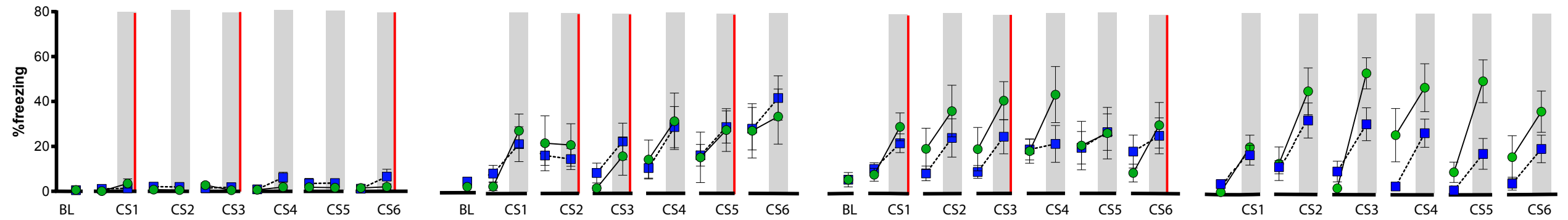

Supplement: S2 Fig — Freezing data are shown for individual trials across the 3 training days and test day for mice trained under reliable and ambiguous cue conditions. Freezing at baseline each day, prior to the first tone, during the tone (gray bars) and 20-sec pre-tone periods are shown. Red lines symbolize shocks that occur at the end of every training trial in the reliable condition and half of the trials in the ambiguous condition; no shocks occurred on the test day. Data are represented as mean ± SEM. (PDF) [file pbio.2001154.s002.pdf]

**A**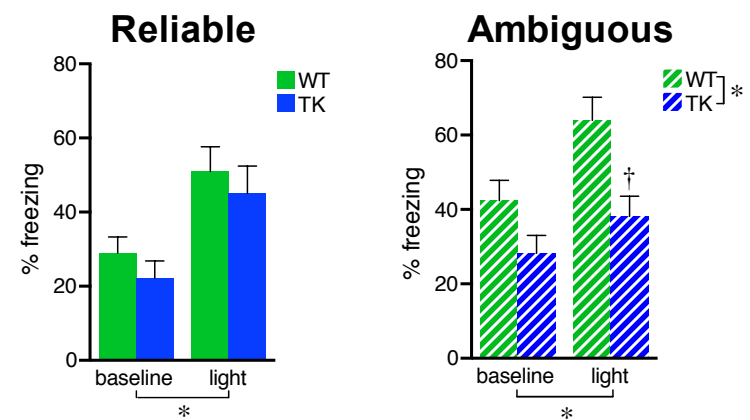**B**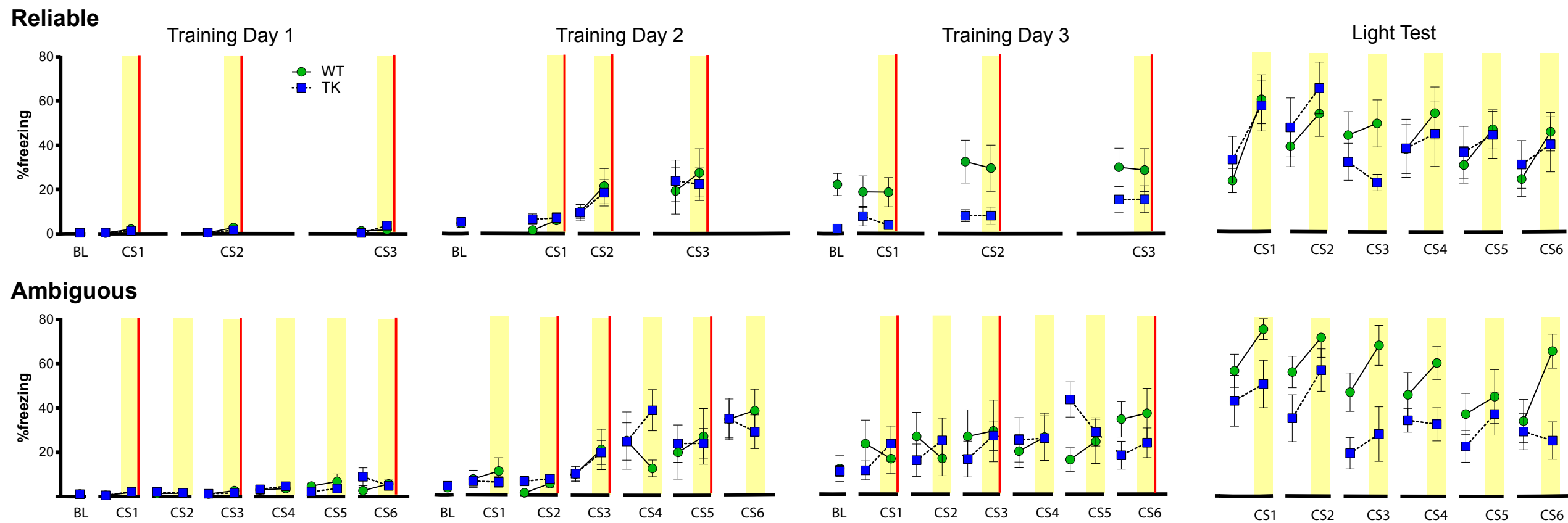

Supplement: S3 Fig — (A) Fear conditioning with a reliable light cue had a similar effect on both genotypes (*, light effect F1,15 = 18.4, p = 0.0007). TK mice froze less than WT mice following ambiguous cued fear conditioning with a light cue (*, light effect F1,15 = 10.4, p = 0.006; genotype effect F1,15 = 9.1, p = 0.009; †, post hoc testing indicates p< 0.05 versus WT at the same time point and condition). In a 3-way ANOVA with cue modality (light/tone), genotype, and predictor (reliable/ambiguous) as factors, the genotype x predictor interaction was significant (F1,56 = 4.6, p = 0.036), with TK mice freezing less than WT mice only after ambiguous conditioning. (B) Freezing data are shown for individual trials across the 3 training days and test day for mice trained under reliable and ambiguous cue conditions. Freezing at baseline each day, prior to the first cue, during the light cues (yellow bars), and during the 20-sec pre-cue periods are shown. Red lines symbolize shocks as in S2 Fig. Data are represented as mean ± SEM. (PDF) [file pbio.2001154.s003.pdf]

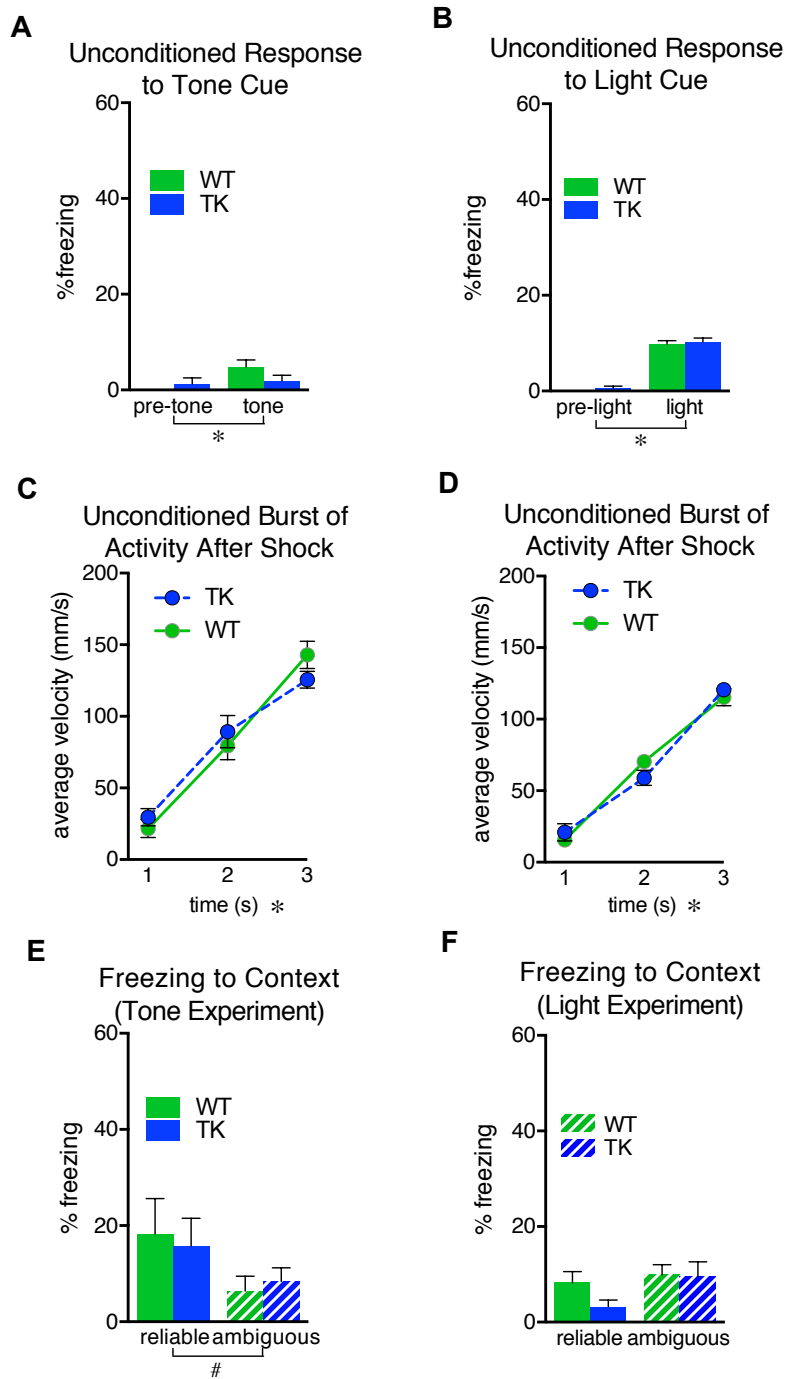

Supplement: S4 Fig — (A) Unconditioned freezing during the first presentation of the tone in tone fear conditioning was similar in WT and TK mice (period, i.e., pre-tone vs. tone, effect F1,27 = 4.9, p = 0.036; no other significant effects). (B) Unconditioned freezing was also similar in WT and TK mice during the first light presentation in fear conditioning to light (period effect F1,33 = 249.4, p<0.0001; no other significant effects). (C) Motor/bursting activity during the first shock did not differ across genotype during tone fear conditioning (time effect: F2,54 = 83.9, p<0.0001; no other significant effects), suggesting similar shock sensitivity. (D) Motor/bursting activity during the first shock also did not differ across genotype during light fear conditioning (time effect: F2,66 = 198.6, p<0.0001; no other significant effects). (E) When tone fear conditioned mice were placed back into the original training context, without cues or shocks, mice in both genotypes showed similar low freezing scores (no significant main effects or interaction; # indicates main effect of predictor type F1,27 = 3.7, p = 0.0654). (F) When light fear conditioned mice were placed back into the original training context, but without cues or shocks, mice in both genotypes showed similar low freezing scores (no significant main effects or interaction). Data are represented as mean ± SEM. (PDF) [file pbio.2001154.s004.pdf]

## Reliable

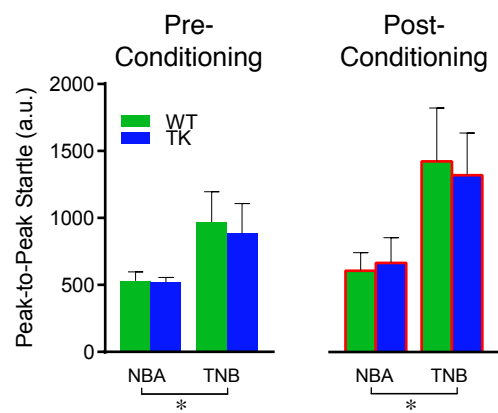

## Ambiguous

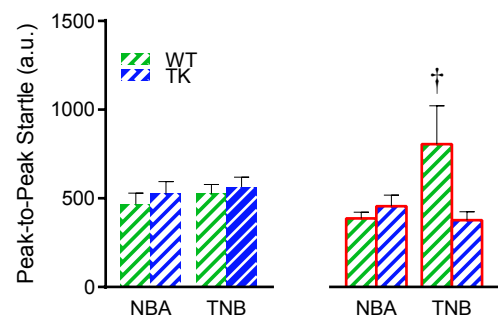

Supplement: S6 Fig — Startle amplitude in arbitrary units (a.u.) is shown for noise burst alone trials (NBA) and for trials in which noise bursts are preceded by the tone cue (TNB). The difference between NBA and TNB reflects potentiation of fear by the cue before fear conditioning (no outline) and after fear conditioning (red outline) with the reliable or ambiguous protocol. In the cohort trained with the Reliable cue, the tone cue increased startle relative to the NBA both pre- and post-conditioning, with no effect of genotype (*, main effect of tone pre: F1,19 = 6.4, p = 0.02, post: F1,19 = 16.3, p = 0.0007; main effect of genotype pre: F1,19 = 0.08, p = 0.78; main effect of genotype F1,19 = 0.004, p = 0.95). In the cohort trained with the Ambiguous cue, there were no significant main effects or interactions prior to conditioning. After conditioning with an ambiguous tone cue, a tone x genotype interaction (F1,19 = 5.4, p = 0.0308; †, post hoc testing indicates p<0.05 versus WT NBA and TK TNB) shows that the tone increased startle in the WT mice but not TK mice. Data are represented as mean ± SEM. (PDF) [file pbio.2001154.s006.pdf]

**A**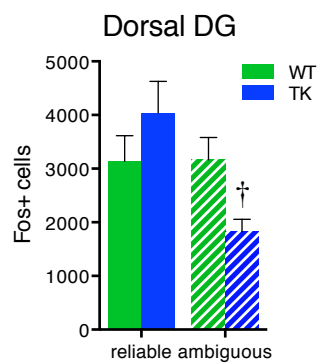**B**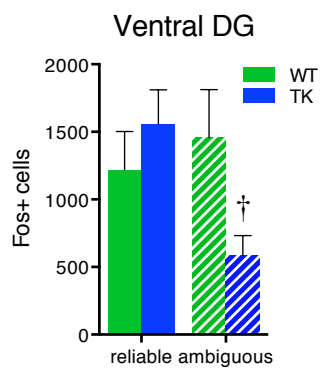**C**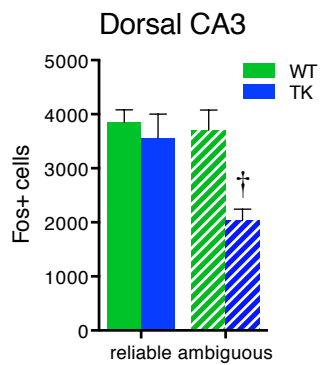**D**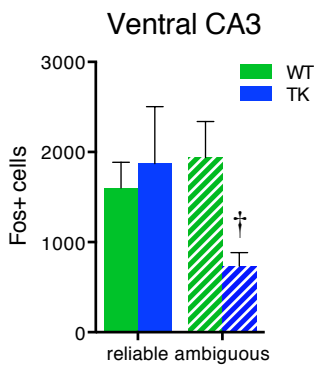**E**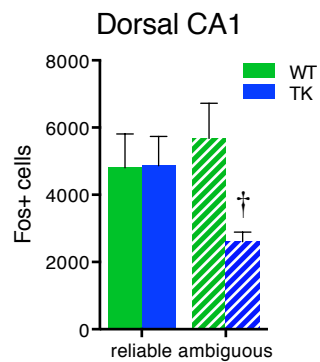**F**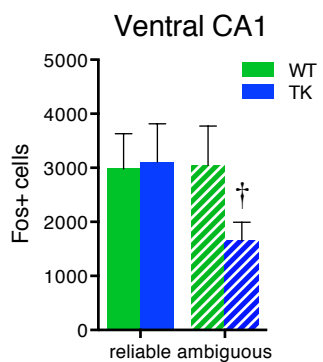

Supplement: S7 Fig — (A) and (B) IEG expression patterns were similar in the dorsal and ventral portions of the dentate gyrus, with fewer Fos+ granule cells in TK mice than WT mice in the ambiguous cue condition but not the reliable cue condition (cue type x genotype interaction: F1,21 = 6.9, p = 0.02 in the dorsal dentate gyrus and F1,21 = 4.7, p = 0.04 in the ventral dentate gyrus; †, post hoc testing indicates p<0.05 relative to WT in the same condition). A three-way ANOVA with cue type, genotype, and region as factors showed a significant cue type x genotype interaction (F1,22 = 6.82, p = 0.02) but no cue type x genotype x region interaction (F1,22 = 2.08, p = 0.16). (C) and (D) IEG expression patterns were also similar in dorsal and ventral CA3, with fewer Fos+ pyramidal cells in TK mice than WT mice after ambiguous, but not reliable, fear conditioning (cue type x genotype interaction: F1,21 = 4.6, p = 0.04 in the dorsal CA3 and F1,21 = 3.9, p = 0.06 in the ventral CA3; †, post hoc testing indicates p<0.05 relative to WT in the same condition). A three-way ANOVA with cue type, genotype, and region as factors showed a significant cue type x genotype interaction (F1,22 = 5.70, p = 0.026) but no cue type x genotype x region interaction (F1,22 = 0.06, p = 0.80). (E) and (F) In the dorsal CA1, TK mice had fewer Fos+ pyramidal cells than WT mice after ambiguous, but not reliable, fear conditioning (cue type x genotype interaction: F1,22 = 3.5, p = 0.07; †, post hoc testing indicates p<0.05 relative to WT in the same condition). In ventral CA1, Fos expression did not show significant main effects (cue type: F1,22 = 1.3, p = 0.27, genotype: F1,22 = 1.1, p = 0.31) or a cue type x genotype interaction (F1,22 = 1.6, p = 0.22; †, post hoc testing indicates p<0.05 relative to WT in the same condition), but the pattern appeared similar to that in the dorsal CA1. A three-way ANOVA with cue type, genotype, and region as factors showed no cue type x genotype interaction (F1,22 = 2.74, p = 0.11) but [file pbio.2001154.s007.pdf]

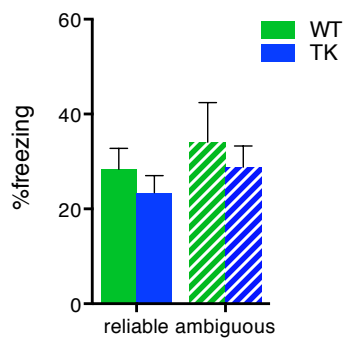

Supplement: S8 Fig — Freezing behavior during the entire 10 min session on the last day of fear conditioning training 2 hr prior to sacrifice was not significantly different across genotype or predictor type, suggesting that differences in behavior during this session did not drive changes in Fos expression. Data are represented as mean ± SEM. (PDF) [file pbio.2001154.s008.pdf]

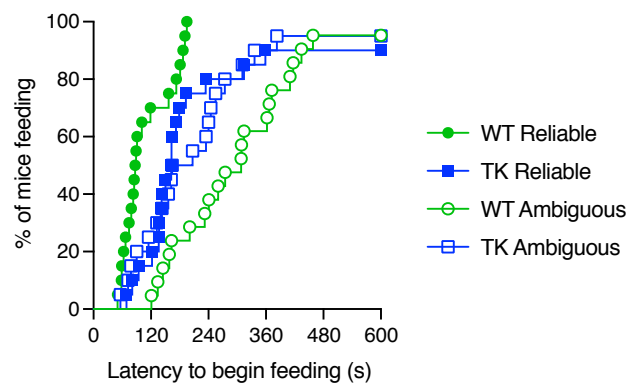

Supplement: S9 Fig — Latency to feed in a novel environment, plotted as mice that have fed at each time point, shows significant differences across genotype/treatment groups (Mantel-Cox test, X2 = 6.255, p = 0.0124). Most mice ate prior to the cutoff of 600s, suggesting that assumptions of normal distribution are not violated, and two-way ANOVA can be used for further analysis. (PDF) [file pbio.2001154.s009.pdf]

Elevated Plus Maze  
Without Fear Conditioning

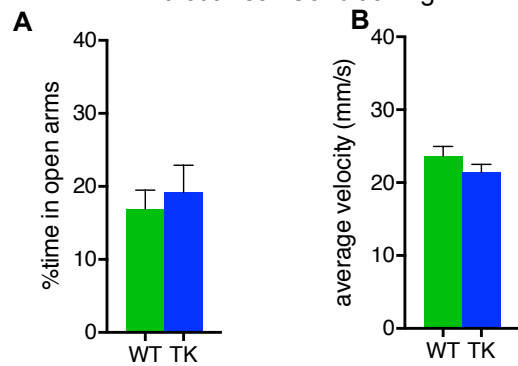

Elevated Plus Maze  
After Fear Conditioning

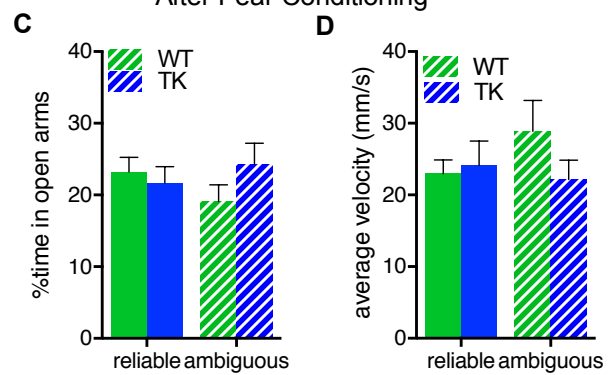

Supplement: S10 Fig — (A) In experimentally naive mice, there was no effect of loss of adult neurogenesis on the percentage of time in open arms of the elevated plus maze. (B) The same mice showed no difference in average velocity during maze exploration. (C) After fear conditioning, a separate cohort of mice showed no effect of predictor type (reliable/ambiguous) or genotype on the percent of time in open arms. (D) The same fear conditioned mice showed no differences in average velocity during exploration. Data are represented as mean ± SEM. (PDF) [file pbio.2001154.s010.pdf]

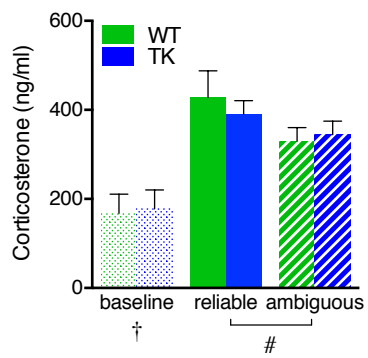

Supplement: S11 Fig — Mice trained on reliable and ambiguous cue fear training both show increased serum corticosterone 30 min after fear conditioning, relative to baseline animals, which had only two days of ambiguous fear conditioning and were tested directly after removal from their home cage (conditioning type: F2,42 = 16.7, p<0.0001; †, post hoc testing indicates p<0.05 relative to treated groups; #, post hoc testing indicates p = 0.0732). (PDF) [file pbio.2001154.s011.pdf]
