# Supplementary material for: Ongoing neurogenesis in the adult dentate gyrus mediates behavioral responses to ambiguous threat cues
Source: PLoS Biol. 2017 Apr 7;15(4):e2001154. doi: 10.1371/journal.pbio.2001154 (PMC5384657; doi:10.1371/journal.pbio.2001154)
Supplement: S5 Fig — Freezing data are shown for individual trials across the 6 days of extinction following reliable cue training/testing. Freezing during baseline (BL) prior to the first tone, during the tones (gray bars), and 20-sec pre-tone periods are shown for each trial. Data are represented as mean ± SEM. (PDF) [file pbio.2001154.s005.pdf]

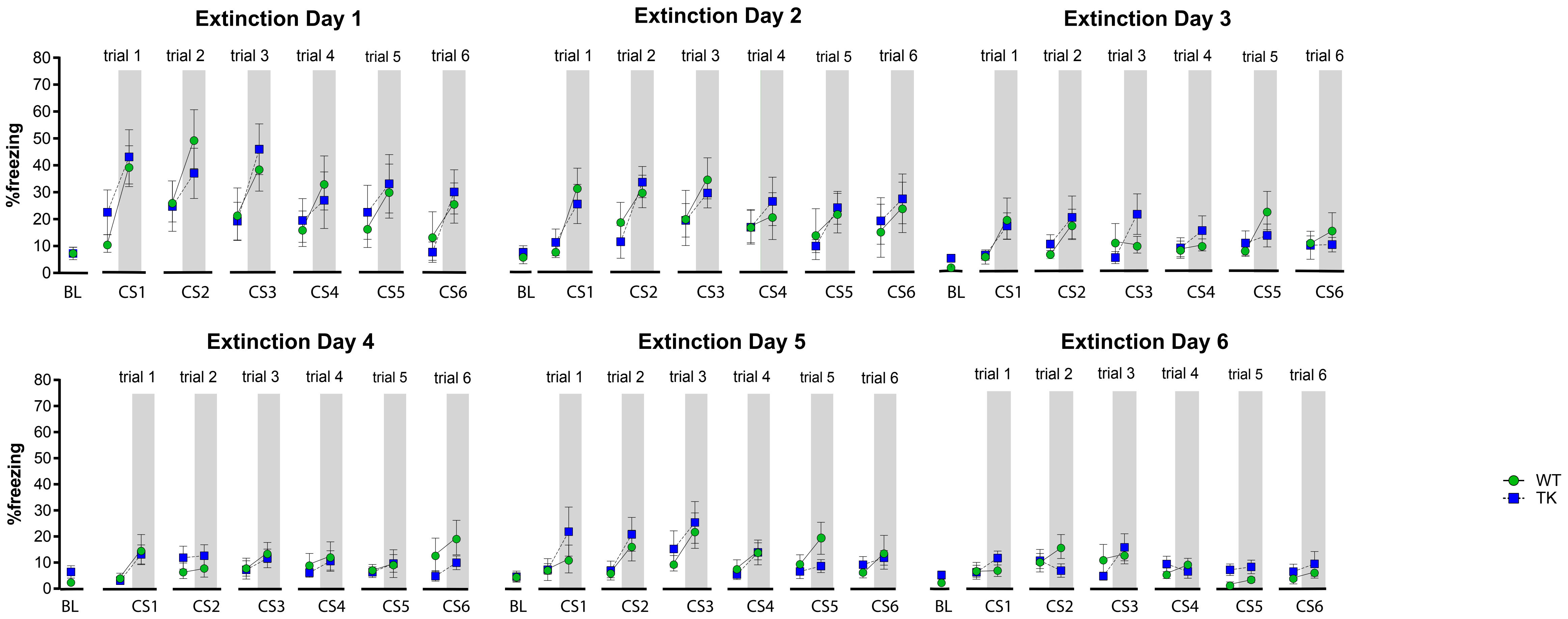
Extinction Day 1
Extinction Day 2
Extinction Day 3
Extinction Day 4
Extinction Day 5
Extinction Day 6
%freezing
0
10
20
30
40
50
60
70
80
trial 1
trial 2
trial 3
trial 4
trial 5
trial 6
BL
CS1
CS2
CS3
CS4
CS5
CS6
WT
TK
